# The Reliability of Red Flags in Spinal Cord Compression

**DOI:** 10.5812/atr.17850

**Published:** 2014-03-30

**Authors:** Nicholas Tobias Johannes Raison, Wisam Alwan, Amit Abbot, Mohamed Farook, Arshad Khaleel

**Affiliations:** 1 Department of Trauma and Orthopaedics, Ashford and St Peter’s NHS Trust, London, England

**Keywords:** Cauda Equina, Spinal Cord Compression, Low Back Pain

## Abstract

**Background:**

Acute low back pain is a common cause for presentation to the emergency department (ED). Since benign etiologies account for 95% of cases, red flags are used to identify sinister causes that require prompt management.

**Objectives:**

We assessed the effectiveness of red flag signs used in the ED to identify spinal cord and cauda equine compression.

**Patients and Methods:**

It was a retrospective cohort study of 206 patients with acute back pain admitted from the ED. The presence or absence of the red flag symptoms was assessed against evidence of spinal cord or cauda equina compression on magnetic resonance imaging (MRI).

**Results:**

Overall, 32 (15.5%) patients had compression on MRI. Profound lower limb neurologic examination did not demonstrate a statistically significant association with this finding. The likelihood ratio (LR) for bowel and bladder dysfunction (sensitivity of 0.65 and specificity of 0.73) was 2.45. Saddle sensory disturbance (sensitivity of 0.27 and specificity of 0.87) had a LR of 2.11. When both symptoms were taken together (sensitivity of 0.27 and specificity of 0.92), they gave a LR of 3.46.

**Conclusions:**

The predictive value of the two statistically significant red flags only marginally raises the clinical suspicion of spinal cord or cauda equina compression. Effective risk stratification of patients presenting to the ED with acute back pain is crucial; however, this study did not support the use of these red flags in their current form.

## 1. Background

Acute low back pain is responsible for a substantial number of primary and secondary care attendances. The majority of current evidence relates to primary care settings where it remains a common presentation (1) with a lifetime prevalence of up to 90% (2). However, literature regarding admission to secondary care is still very limited. Although benign causes account for approximately 95% of back pain (3), the potential severity of the remaining causes necessitates timely and accurate diagnosis.

Although the acute spinal cord and cauda equina compression are rare, the consequences of such serious spinal pathologies are devastating. Incidence ranges between one to eight in 100000 (4). These complex neurological disorders present with a myriad of symptoms. The most common etiologies are lumbar disc herniation, spinal stenosis, and tumors (both primary and metastatic) (4, 5). Rapid diagnosis is vital with delay leading to increased risk of morbidities and disability (6, 7). Meta-analysis of cauda equina syndrome has shown the benefit of as early decompression as within 48 hours of the onset of symptoms (8).

Magnetic resonance imaging (MRI) is the gold standard investigation tool in non-traumatic injuries (9-11). In contrast, for patients with uncomplicated back pain, routine imaging has been shown to have no impact on outcome (12, 13) and even can be detrimental (14). Therefore, risk stratifying is crucial and consequently, clinical guidelines have been developed (15) that use red flag signs to attempt to highlight these patients. Various red flag signs and symptoms are routinely used in the assessment of low back pain to identify spinal cord emergencies. These can be divided into three main categories: spinal fractures, malignancy or serious inflammatory conditions, and spinal cord compression or cauda equina.

The red flags used most commonly for identifying spinal cord compression, irrespective of the etiology, are profound motor or sensory weakness in the lower extremities, bowel or bladder dysfunction, and saddle distribution sensory disturbance (16). The presence of any of them raises the suspicion of cord compromise and prompts further investigation and specialist intervention. However, selection of these particular red flags has largely been based on inference with little rigorous data supporting their prognostic capability (17). Additionally, no studies have been performed to date to look at their effectiveness in differentiating low back pain in the context of acute presentations to the emergency department (ED).

As a standard practice, it falls to the clerking junior doctor in ED to identify such red flags and this study aimed to evaluate their prognostic value at this point. In line with international guidelines, the assessed red flag symptoms were bowel and bladder dysfunction, saddle sensory disturbance, and widespread or progressive weakness in legs or gait disturbance.

## 2. Objectives

The aim of our study was to investigate the association between the presence of red flag symptoms and radiologically proven spinal cord or cauda equina compression.

## 3. Patients and Methods

A retrospective cohort study of patients attending the ED of a district general hospital was conducted. Patients were identified from the electronic admission records with a primary complaint coded as “back pain”. The hospital notes were then obtained and reviewed. Imaging reports were taken from the Picture Archiving and Communications System (PACS) radiology database and electronic pathology reports.

All adult patients presenting to ED with back pain that warranted a referral to the orthopaedic team between January 2004 and December 2008 were identified. Patients diagnosed with traumatic or degenerative spinal fractures were excluded. Out of the 239 patients, the case notes for 206 were retrieved and analyzed. Symptoms at presentation together with the examination findings, specifically those of spinal and neurological assessment, were considered from the case notes. The assessment and presence of red flag symptoms at presentation was recorded in all patients. The results of investigations undertaken during that admission and all radiological studies were analyzed. A consultant radiologist reported all MRI scans. Those showing compression of the spinal cord and cauda equina were labelled as positive result. Pearson’s Chi^2^ test was used to determine the association between the individual red flag symptoms and a positive MRI result. For those red flags that were found to have a significant association, further analysis was performed to assess their prognostic potential. The 2×2 tables were used to calculate the sensitivity, specificity, and the likelihood ratios.

## 4. Results

Of the 206 patients who presented with back pain, 32 (15.5%) had compression of the spinal cord or cauda equina on MRI. All patients were assessed by the general orthopedic registrar on-call. Thirty patients had some disturbance of saddle sensation with 11 (36.7%) cases of spinal cord compression. Thirty-nine patients were found to have profound lower limb neurological dysfunction, but only 9 (23%) patients had a positive MRI finding. Finally, of the 65 patients with disturbance of urinary and/or bowel sphincters, 18 (27.7%) patients had MRI proven spinal cord compression.

The results from the Pearson’s Chi^2^ test gave high χ2 statistics for bowel/bladder disturbance and saddle sensory dysfunction, 10.70 (P = 0.001) and 11.60 (P = 0.0005), respectively, suggesting a significant association with spinal cord compression. However, there was no significant association with profound lower limb neurology, χ^2^ = 2.09 (P = 0.15). The value of these two clinical red flags as markers for cord compression was assessed further (Table 1). The positive likelihood ratios demonstrated that two of the red flag symptoms (bowel/bladder disturbance and saddle sensory dysfunction) were positively associated with cord compression. However, the relatively modest figures indicated that this diagnostic effect was small, even when the two symptoms were taken in conjunction.

**Table 1.** Statistical Analysis of Red Flag Symptoms ^a^

Symptom	Sensitivity	(95% CI)	Specificity	(95% CI)	Likelihood Ratio
**Saddle sensory disturbance **	0.27	(0.12 - 0.48)	0.87	(0.81 - 0.92)	2.11
**Bowel/bladder dysfunction**	0.65	(0.44 - 0.82)	0.73	(0.66 - 0.80)	2.45
**Saddle sensory disturbance or bowel/bladder dysfunction**	0.27	(0.12 - 0.48)	0.92	(0.87 - 0.96)	3.46

^a^ Abbreviation: CI, confidence interval.

## 5. Discussion

Of the three symptoms routinely given as red flags for cord compression (16), the results from this study demonstrated that only two, namely bowel/urinary symptoms and saddle sensory disturbances, had a significant association with positive findings in MR imaging. Their corresponding likelihood ratio (LR) showed that their presence in back pain had increased the risk of cord compression. Yet, this predictive value was modest and only marginally raised the clinical suspicion of a cord compression rather than providing definitive support of the diagnosis. The high specificity and low sensitivity showed that the symptoms were better for excluding compression rather than identifying it.

The focus of this study was the identification of the red flags by the junior doctors in the ED. Eliciting such signs and symptoms is dependent on the expertise of the examiner. It is, therefore, not surprising that large discrepancies were noted in their recognition and documentation. This operator bias helps to explain the poor prognostic ability of the red flags. For not only were the symptoms often subtle but a patient’s past medical history and comorbidities could further obscure the picture (18). Other confounding factors that were noted during data collection, common in the ED, included symptomatic bladder outflow obstruction, pelvic floor dysfunction, and UTI. Pain in itself can also greatly impede an accurate assessment. Similar problems were highlighted in previous studies looking at the predictive value of a neurosurgery registrar’s examination (19). The authors highlighted a common conundrum of back pain with concurrent sciatica, with pain causing the difficulty in micturition rather than any cord compromise.

The subtlety of the early symptoms of cord compression is another important factor. The classic presentation of a patient with back pain, weakness, and true acute urinary retention is rare. Our results showed that the majority of the patients presented with a few largely equivocal signs. Such complexity and variation in neurological symptoms was previously established *in vivo*. Studies using identical selective nerve blocks to reproduce radicular symptoms found varying dermatomal distribution of numbness between individuals (20). Cauda equina itself manifests in a variety of ways from an acute event to more chronic progressive disease (6) with differing symptom patterns. Such poor predictive potential of the red flag symptoms has significant implications for clinical practice. The resultant diagnostic uncertainty in patients presenting to the accident and emergency department with low back pain is clearly highlighted. Given the severity of the disease, the need to make a fast diagnosis, and the lack of availability of MRI, particularly out of hours, red flags clearly have an important role. However, in their current form, this study did not demonstrate their value in the clinical setting.

Neurosurgery and neurology, in general, are only briefly covered in undergraduate curricula (21) and consequently, teaching on the recognition of neurological emergencies such as acute cord compression is limited. The main aim for junior clinicians is to recognize and highlight potentially significant diseases. Therefore, the tendency to simplify situations to the recognition of a few red flags is understandable but, as this study showed, not effective. Even the basic test of Babinski’s reflex, taught to medical students worldwide, has been shown to be a relatively poor marker for central nervous system lesions (22).

Therefore, while red flags may remain important clinical markers, the degree of suspicion in such patients needs to be very low. Although the prognostic power of the red flags is poor, it is still positive. An active process of exclusion of these symptoms rather than a passive acknowledgment of their presence is needed. Effective risk stratification of patients presenting to the ED with acute back pain is crucial for appropriately directing patients to the correct specialist services. Use of the red flag signs to aid this process is clearly important, but standardization of eliciting them effectively and appropriate education regarding their diagnostic value is essential. Given the significant morbidity and mortality of cord compression, there should be a high degree of suspicion when the signs are present while a low threshold for imaging of the spine. This study has highlighted the need for further investigation on more sensitive diagnostic tests and criteria to better identify those patients at risk of cord compression.
